# Relationship Between Internet Use Purposes and Depressive Symptoms Among Chinese Older Adults: A Cross‐Lagged Panel Network Analysis

**DOI:** 10.1155/da/3715228

**Published:** 2026-05-09

**Authors:** Yan Wu, Yang Yang

**Affiliations:** ^1^ Department of Thoracic Surgery, Nanjing Chest Hospital, Affiliated Nanjing Brain Hospital, Nanjing Medical University, Nanjing, 210029, Jiangsu, China, njmu.edu.cn

**Keywords:** cross-lagged panel network, depressive symptoms, internet use purposes, older adults

## Abstract

**Background:**

There were debates in the results about the complex associations between Internet use purposes and depressive symptoms. A key limitation of prior research is its treatment of depressive symptoms as a unitary construct, which neglects the dynamic and specific interactions between different online activities and distinct depressive symptoms. Therefore, this study aimed to explore the relationships between Internet use purposes and depressive symptoms by cross‐lagged panel network (CLPN) analysis.

**Methods:**

Data comes from two waves of the China Health and Retirement Longitudinal Study (CHARLS; 2018 and 2020). Internet use purposes were assessed by a single item. Depressive symptoms were assessed by the Center for Epidemiological Studies Depression Scale (CES‐D). The CLPN analysis was used to explore the dynamic network interactions between Internet use purposes and depressive symptoms.

**Results:**

We included a total of 9290 older adults. A higher level of “Watching news” (In2) can predict lower level of multiple depressive symptoms, such as “Everything was an effort” (De4) (*β* = −0.19), “Feeling hopeful about the future” (De5) (*β* = −0.16). “Chatting” (In1) predicted lower “Lack of happiness” (De8) (*β* = −0.09), and “Playing games” (In4) predicted lower “Everything was an effort” (De4) (*β* = −0.12). In contrast, “Watching videos” (In3) predicted higher “Everything was an effort” (De4) (*β* = 0.09), and “Financial management” (In5) predicted higher “Lack of happiness” (*β* = 0.14). In addition, “Lackness of happy” (De8) was had higher out expected‐influence (Out‐EI) and in expected‐influence (In‐EI) of the node.

**Conclusion:**

This study indicated that different Internet use purposes had distinct and specific associations with specific depressive symptoms among older adults. These findings underscore the necessity for future interventions to move beyond general Internet use and instead digital strategies designed to address particular depressive symptoms.

## 1. Introduction

With the rapid growth of Internet use among Chinese older adults [1], the impacts of different Internet use purposes on specific depressive symptoms have also been widely examined in this population [2, 3], but the results remain debated. For example, Lam et al. [4] reported that obtaining Internet information resulted in the low level of individual life satisfaction. In contrast, Fan et al. [2] found that watching news, watching videos, and playing games may alleviate depressive symptoms, while online chatting is not related to depressive symptoms. These inconsistent findings may challenge both researchers and clinical practitioners in determining how to encourage older adults to use the Internet in order to improve depressive symptoms.

A potential source of the controversial results may lie in the data analytical methods. Prior studies often assess depressive symptoms by the mean or total score of its symptoms [5], analyzing its relationship with Internet use purpose via linear regression. This approach overlooks that each symptom of depressive symptoms is unique and not equivalent [6] and covers the complex interaction between Internet use purposes and specific depressive symptoms, potentially leading to mixed contradictory results.

Cross‐lagged panel network (CLPN) model provides a novel framework to address the above problems. It conceptualizes Internet use purposes and depressive symptoms as systems of interacting components [7]. In such a network, components of depressive symptoms and multiple Internet use purposes are regarded as nodes, and their statistical relationships are modeled as edges. CLPN extends this framework longitudinally, enabling the exploration of temporal and potentially bidirectional relationships between specific Internet use purpose and specific depressive symptoms over time [8]. However, no prior study has applied CLPN to investigate the dynamic relationships between specific Internet purposes and depressive symptoms in older adults. Therefore, this study aims to employ CLPN to map these complex associations in a national sample of Chinese older adults. Based on the above analysis, we hypothesize that different types of internet use purposes have different effects on specific depressive symptoms.

## 2. Method

### 2.1. Participants

We adopted data from the China Health and Retirement Longitudinal Study (CHARLS), which employed a stratified sampling design to survey participants aged 45 and over in China, ensuring broad geographical and demographic representation [9]. So far, five surveys have been conducted, namely those in 2011, 2013, 2015, 2018, and 2020. The database has been published on the CHARLS website (http://charls.pku.edu.cn), which is accessible following registration and permission. Before this study began, all participants provided informed consent.

**Figure 1:**
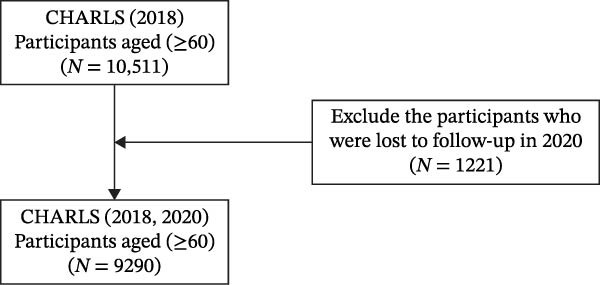
The flowchart of participants selection.

Since only the surveys in CHARLS from 2018 and 2020 measured Internet use purposes, we adopted the data from these two waves in this study. The included criteria were (1) aged 60 years or older in 2018 and (2) completed assessments of depressive symptoms and Internet use purposes in 2018 and 2020. We finally included 9290 participants. The detailed process of sample selection is illustrated in Figure 1.

### 2.2. Measure

#### 2.2.1. Internet Use Purposes

A single‐item was used to assess Internet use purposes of participants. Participants responded to the question: “What do you usually do on the Internet?” The answers included “Chatting,” “Watching news,” “Watching videos,” “Playing games,” and “Financial management.” If the respondent selects a certain item, it is assigned as 1, otherwise it is assigned as 0.

#### 2.2.2. Depressive Symptoms

The Center for Epidemiological Studies Depression Scale (CES‐D) was used to evaluate depressive symptoms [10]. This scale consisted of 10 items and adopted a 4‐point Likert scale. 0 indicated rarely or none of the time; 3 represented most or all of the time. Among these items, items 5 and 8 were scored in reverse. The total score ranged from 0 to 30 points, with higher scores indicating more severe depressive symptoms. A cut‐off of ≥10 suggested clinically relevant depressive symptoms [11].

#### 2.2.3. Covariates

Covariates included socioeconomic status, health‐related factors, and digital literacy. Socioeconomic status included gender (male/female), age, and education level (illiterate/literate). Health‐related factors included chronic disease history. Digital literacy included types of devices for accessing the Internet and the frequency of Internet use. Age is the age of the participants in 2018, and the calculation method is the survey time minus the actual birth time of the participants.

### 2.3. Data Analysis

As shown in Table S1. The missing rate of data ranged from 9.70% to 27.03%. To explore the mechanism of missing data, we created a missingness matrix and assessed correlations between missing values (Table S2). Strong associations in missing values among specific depressive symptoms suggested systematic patterns, suggesting that these data were possibly missing at random (MAR). Therefore, these missing values were handled via Multiple Imputation by Chained Equations (MICE) in R 4.3.1 [12]. In contrast, missing data for education level showed weak correlations with other variables, consistent with a pattern of missing completely at random (MCAR). Due to its low proportion and independence, observations with missing values for this variable were directly excluded from subsequent analyses. Given the level of missingness (<30%) for some variables, five imputed datasets with 10 iterations following previous studies [13, 14]. Because our study focuses on the structural pattern of the cross‐lagged network (i.e., the presence/absence and relative strength of relationships) rather than on statistical inference (e.g., confidence intervals [CIs] or hypothesis tests), the point‐estimation step of Rubin’s rules was used to combine the results from the five imputed datasets (refer to Tables S3 and S4). For each of the five imputed datasets, we independently estimated the cross‐lagged network matrix using elastic net regression, as implemented in the R package cv.glmnet. The regularization parameter *λ* was selected by minimizing the cross‐validation error. This procedure yielded a 15 × 15 network matrix per dataset. We then averaged the corresponding elements of these five matrices to obtain the final combined network matrix.

Descriptive analysis was conducted by using SPSS 25.0. Age was described by mean and standard deviation, given its normal distribution (skewness = 0.157; kurtosis = −1.976). Frequencies and proportions were used to describe the remaining categorical variables. The variance inflation factor was used to detect multicollinearity among independent variables.

The CLPN was conducted by R 4.3.1, which analyzes dynamic relationships between Internet use purposes and depressive symptoms over time [15]. Least absolute shrinkage and selection operator (LASSO) was used to construct CLPN via R package glmnet [16]. In LASSO regression, the regularization parameter (*λ*) was strictly selected through 10‐fold cross‐validation, with the aim of minimizing the cross‐validation prediction error (mean squared error [MSE]) [17]. The standard LASSO penalty (*α* = 1) was used to promote sparsity and enhance interpretability of the network structure. The standardized variables were adopted to ensure comparability of regularization across different predictors. The CLPN comprised autoregressive effects and cross‐lagged effects. The former indicates the influence of a node on itself over time. The latter suggests the impact of a node in 2018 on other nodes in 2020. The qgraph package was adopted to visualize the CLPN. Nodes indicate items, and arrows suggest directions of the associations between different nodes. Blue arrows indicate positive relationships, and red arrows indicate negative relationships. The thicker the line was, the stronger the relationship between the nodes was. Out expected‐influence (Out‐EI), in expected‐influence (In‐EI), and bridge expected‐influence (Bridge‐EI) were adopted to estimate variable centrality. Network stability and accuracy were estimated using the bootnet package. The stability of the networks was quantified by correlation stability coefficients (CS‐coefficient), According to Epskamp et al. [18], CS‐coefficients above 0.25 are considered the minimum acceptable level for interpretable networks, whereas values above 0.5 are preferred. The robustness of our temporal inferences was evaluated by integrating this index with the bootstrapped 95% CIs of edge weights for network, and bootstrapped difference tests of centrality and edge weights. The edge was considered to be reliable when the bootstrapped 95% CI of an edge weight was narrow.

## 3. Result

### 3.1. Participant Characteristics

We finally included a total of 9290 participants. The mean age of participants was 68.81 (SD = 6.82) years; 48.5% were male; more than half received no formal education (53.9%); 457 participants only use one device to access the internet. 487 participants access the internet almost daily. Table 1 presents the items’ information for Internet use purposes and depressive symptoms. As shown in Table S5, the multiple collinearity test results show that the VIF values of all variables are all less than 5, indicating that there is no multicollinearity among the variables.

**Table 1:** The items information for the Internet use purposes and depressive symptoms.

Variables	*N* (%) (T1)	*N* (%) (T2)
Depressive symptoms		
Unusually bothered	4230 (45.5)	4572 (49.2)
Mind adrift	4435 (47.7)	4745 (51.1)
Felt depressed	4499 (48.4)	4486 (48.3)
Everything was an effort	3996 (43.0)	4726 (50.9)
Felt hopeful about the future	5970 (64.3)	6410 (69.0)
Felt fearful	1956 (21.1)	2083 (22.4)
Restless sleep	4963 (53.4)	4869 (52.4)
Lack of happiness	4851 (52.2)	5247 (56.5)
Loneliness	2968 (31.9)	2936 (31.6)
Inability get going	2276 (24.5)	2414 (26.0)
Internet use purposes		
Chatting	341 (3.7)	1035 (11.1)
Watching news	466 (5.0)	1365 (14.7)
Watching videos	368 (4.0)	1336 (14.4)
Playing games	144 (1.6)	233 (2.5)
Financial management	44 (0.5)	77 (0.8)

*Note: N* represents frequency, % represents proportion.

### 3.2. Temporal Network

**Figure 2:**
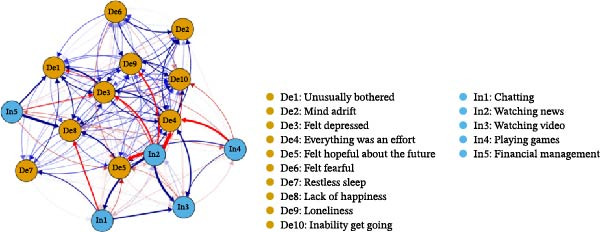
The temporal network of Internet use purposes and depressive symptoms. The autoregressive effects and |*β*| < 0.05 were excluded in order to visual demonstration of the temporal relationships.

The adjacency matrix of the temporal network is presented in Tables S3 and S4. Figure S5 shows all directed edges in network graph. Figure 2 excluded autoregressive effects and weaker connections (|*β*| < 0.05) in order to visual demonstration of the temporal relationships.

A higher level of “Chatting” (In1) in 2018 was related to lower level of “Lack of happiness” (De8) (*β* = −0.09) in 2020. “Watching news” (In2) at 2018 was negatively associated with “Everything was an effort” (De4) (*β* = −0.19) and “Feeling hopeful about the future” (De5) (*β* = −0.16) in 2020. “Watching videos” (In3) at 2018 was positively related to “Everything was an effort” (De4) (*β* = 0.09) in 2020. “Playing games” (In4) in 2018 was negatively associated with “Everything was an effort” (De4) (*β* = −0.12) in 2020. “Financial management” (In5) in 2018 predicted a higher future level of “Lack of happiness” (De8) (*β* = 0.14) in 2020, representing the single strongest effect in the temporal network. All other predictive paths were weak (|*β*| ＜ 0.09).

**Figure 3:**
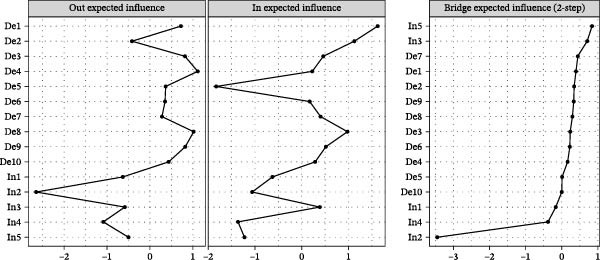
The out expected‐influence, in expected‐influence, and bridge expected‐influence in the temporal network. De, depressive symptoms; In, internet use purposes.

In‐EI and Out‐EI are shown in Figure 3. The three highest In‐EI of the nodes were “Unusually bothered” (De1), “Mind adrift” (De2) and “Lack of happiness” (De8), suggesting they were most easily predicted by other nodes. The three highest Out‐EI were “Everything was an effort” (De4), “Lack of happiness” (De8) and “Felt depressed” (De3), indicating they were more able to predict other nodes. The highest Bridge‐EI of node was “Financial management” (In5), indicating it was the core nodes in the relationship between specific Internet use purpose and depressive symptoms.

The CS coefficients for In‐EI, Out‐EI, and Bridge‐EI are 0.439, 0.281, and 0.750, respectively (refer to Figure S2). Based on the guidelines proposed by Epskamp and Fried [18], this value falls within the acceptable range (>0.25) but does not reach the more stringent threshold (>0.5), indicating that the stability of the expected influence indices is acceptable but not optimal. Figure S1 shows the bootstrapped 95% CIs of edge weights. The difference tests of centrality and edge weights are shown in Figures S3 and S4.

## 4. Discussion

To our knowledge, this is the first study to investigate the dynamic relationship between specific Internet use purposes and depressive symptoms within a Chinese representative sample of older adults via CLPN. Consistent with our hypothesis, the study found that “Watching news” (De2) in 2018 negatively predicted multiple depressive symptoms in 2020, while other Internet use purposes in 2018 exhibited complex dual associations with components of depressive symptoms in 2020.

### 4.1. The Positive Effects of Internet Use Purposes on Depressive Symptoms

In the temporal network, higher “Watching news” (In2) was most strongly associated with lower level of “Everything was an effort” (De4) and a higher level of “Feeling hopeful about the future” (De5) among older adults. Previous studies have indicated that watching news fosters a sense of connection to the wider world and can distract from daily worries, potentially enhancing mood and reducing fatigue [2].

### 4.2. The Dual Effects of Internet Use Purposes on Depressive Symptoms

In contrast, other Internet use purposes had dual effects on multiple depressive symptoms. “Chatting” (In1) was the best predictor of “Lack of happiness” (De8) and “Feeling hopeful about the future” (De5). “Chatting” (In1) can expand older adults’ social networks by overcoming constraints of time and space. This expansion can alleviate loneliness while enhancing social support, thereby contributing to improved psychological well‐being and greater confidence in facing daily life [2, 19, 20]. However, “Chatting” (In1) also showed a weak positive correlation with “Restless sleep” (De7), which may be related to the fact that the excessive duration of online chatting exceeds older adults’ physical tolerance range [21]. Therefore, older adults should do online chatting appropriately to prevent excessive use from causing cognitive overload, social substitution, and physiological interference, which in turn may have a negative impact on their psychological well‐being.

We also found that “Watching videos” (In3) was most strongly related to a high level of “Everything was a effort” (De4), while it had negative association with “Feeling hopeful about the future” (De5). Previous studies have also found that older adults can obtain various types of information through video content, which expands their knowledge, meets their spiritual and cultural needs, enriches their lives, and thus promotes happiness and feelings of hope about the future [22]. However, this type of information acquisition via the Internet can take up the time that older adults spend interacting with others and meets some of their needs that would require help from others. Consequently, the opportunities and necessity of older adults for face‐to‐face social interaction are reduced, potentially increasing feelings of loneliness and diminishing confidence [3]. At the same time, excessive indulgence in virtual life can lead to physical and mental troubles, such as visual impairment and cognitive impairment, and increase the feelings of fatigue [23]. Therefore, when older adults engage in online entertainment activities, they should aim to seek benefits and avoid harms in order to fully enjoy the advantages of digital life.

“Playing games” (In4) was most strongly related to a low level of “Everything was a effort” (De4), indicating that gaming had potential to help older adults to divert their attention from annoying matters and relieve fatigue [24]. However, “Playing games” (In4) was positively associated with “Feeling hopeless about the future” (De5), suggesting that prolonged or frequent gaming may contribute to increased hopelessness, or that hopeless individuals may increasingly turn to gaming as an escape. These bidirectional possibilities highlight the complex role of gaming: while it may offer short‐term relief from daily exertion, it may also reinforce maladaptive cognitive patterns related to future expectations.

“Financial management” (In5) was negatively correlated with “Mind adrift” (De2) and “Feeling depressed” (De3), whereas it was most strongly associated with an increased “Lack of happiness” (De8). Moreover, “Financial management” (In5) was the node with the highest Brigde‐EI. Following retirement and the attendant weakening of social roles, managing finances can become a key domain of personal control for older adults, serving as a way to counteract feelings of powerlessness associated with aging. However, it often provides a sense of confidence rather than happiness. This is partly because the process involves both risk and return, with older adults typically focusing more on potential losses and minutiae (e.g., fees, account fluctuations) than on gains, and neuroscientific evidence suggests they exhibit heightened neural sensitivity to financial losses alongside a dampened response to gains [25].

### 4.3. Centrality of “Lack of Happiness”

“Lackness of happiness” (De8) was higher Out‐EI and In‐EI of the node. Prior studies suggested that “Lack of happiness” (De8) was the core symptom of depressive symptoms and influence other depressive symptoms [26]. All purposes of Internet use were correlated with “Lack of happiness” (De8) in this study. This finding was consistent with previous research findings [27] and indicated “Lack of happiness” (De8) could be a potential intervention target for alleviating depressive symptoms through guided Internet use.

### 4.4. Clinical Implications

These findings argue against one‐size‐fits‐all digital guidance. Practical implications are as follows. First, encouraging moderate news consumption can be promoted as a low‐threshold intervention to foster connectivity and hope. Second, guiding balanced use of chatting, watching videos, and playing games can maximize benefits and minimize risks like sleep disruption. Third, particular attention should be paid to financial management, as its high bridge centrality identifies it as a potent pathway influencing mood, warranting education on mindful use. Finally, efforts should be redirected from monitoring online activities themselves to managing the specific “behavior‐emotion” pathways that influence this core symptom, such as “Lack of happiness” (De8). This approach regards digital technology as a positive resource rather than a mental health risk for older adults.

### 4.5. Limitation

Although this study has strengths, its limitations should also be acknowledged. First, this study adopted self‐report measures rather than clinical diagnoses for depressive symptoms. Therefore, it’s difficult to avoid self‐report bias and to reflect the real relationship between Internet use purposes and depressive symptoms in a clinical diagnostic sample. Future studies should investigate this relationship through clinical diagnostic criteria. Second, CLPN only explores the associations between variables. Hence, the results of this study can be regarded as hypotheses, but cannot establish causation. In addition, the two‐wave design does not enable us to explore bidirectional relationships within a more precise timeframe. Future research should use multiple waves of data. The current study utilized only two waves of CHARLS data (2018–2020), which limits our ability to establish stable bidirectional or causal relationships. Third, although the CS‐coefficient for Out‐EI and In‐EI reached the minimum acceptable level of 0.25 as proposed by Epskamp and Fried [18], it did not achieve the more stringent threshold of 0.5 that is often recommended. This finding suggests that the estimates of node importance, particularly the rankings of Out‐EI and In‐EI, may be relatively sensitive to sample size. Future studies with larger samples are warranted to validate the robustness of these centrality estimates and to obtain more stable network structures. Finally, Internet use purposes were measured using binary (yes/no) responses, which may oversimplify these multidimensional behaviors. This measurement approach reduces variability in responses and may not capture the frequency, intensity, or context of Internet use. Future research would benefit from employing more nuanced measures, such as frequency scales, time‐use diaries, or qualitative assessments, to better capture the multidimensional nature of Internet use behaviors.

## 5. Conclusion

This study revealed complex and bidirectional correlations between specific Internet use purposes and depressive symptoms among older adults. Findings demonstrated that “Watching news” (In2) has the potential to alleviate future depressive symptoms, while other Internet use purposes have dual effect on specific depressive symptoms. “Lackness of happy” (De8) may serve as a target for various Internet‐based interventions for depressive symptoms.

**Nomenclature**
CLPN: Cross‐lagged panel network analysis
CHARLS: China Health and Retirement Longitudinal Study
CES‐D: Center for Epidemiological Studies Depression Scale
LASSO: Least absolute shrinkage and selection operator
Out‐EI: Out expected‐influence
In‐EI: In expected‐influence
Bridge‐EI: Bridge expected‐influence
CI: Confidence interval.

## Author Contributions

Yan Wu completed the following tasks of this article, including conceptualization, data interpretation, methodology, formal analysis, visualization, writing, and reviewing. Yang Yang completed the following tasks of this article, including conceptualization, data interpretation, methodology, supervision, writing, reviewing, and editing.

## Funding

This work was supported by the General Project of Nanjing Medical University, Science and Technology Development Fund in 2024 (Grant NMUB20240216).

## Disclosure

All the authors have approved the final manuscript.

## Ethics Statement

The study was conducted in accordance with the principles of the Helsinki Declaration and approved by the Biomedical Ethics Review Committee of Peking University (IRB00001052‐11015). All participants signed informed consent before conducting the surveys.

## Conflicts of Interest

The authors declare no conflicts of interest.

## Supporting Information

Additional supporting information can be found online in the Supporting Information section.

## Supporting information


**Supporting Information** A supplementary file shows more details of the data analysis in this article (refer to supplementary material). Table S1: Missing frequency and Percentage for a dataset of 9290 participants. Table S2: Correlation matrix of missing data patterns. Table S3: Adjacency matrix of the temporal network (including autoregressive edges). Table S4: Adjacency matrix of the temporal network. Table S5: Multicollinearity diagnostics of variables. Figure S1: Bootstrapped 95% confidence intervals around temporal network edges. Figure S2: Centrality of the temporal network. Figure S3: Centrality of difference tests for the temporal network. Figure S4: Edge weight difference tests for the temporal network. Figure S5: The cross‐lagged panel network analysis of internet use purpose and depressive symptoms (including autoregressive edges).

## Data Availability

This study used open data. The data are publicly available in CHARLS at http://charls.pku.edu.cn/en.
