# Supplementary material for: Relationship Between Internet Use Purposes and Depressive Symptoms Among Chinese Older Adults: A Cross‐Lagged Panel Network Analysis
Source: Depress Anxiety. 2026 May 9;2026:3715228. doi: 10.1155/da/3715228 (PMC13157306; doi:10.1155/da/3715228)
Supplement: Supplementary file 1 — Supporting Information A supplementary file shows more details of the data analysis in this article (refer to supplementary material). Table S1: Missing frequency and Percentage for a dataset of 9290 participants. Table S2: Correlation matrix of missing data patterns. Table S3: Adjacency matrix of the temporal network (including autoregressive edges). Table S4: Adjacency matrix of the temporal network. Table S5: Multicollinearity diagnostics of variables. Figure S1: Bootstrapped 95% confidence intervals around temporal network edges. Figure S2: Centrality of the temporal network. Figure S3: Centrality of difference tests for the temporal network. Figure S4: Edge weight difference tests for the temporal network. Figure S5: The cross‐lagged panel network analysis of internet use purpose and depressive symptoms (including autoregressive edges). [file DA-2026-3715228-s001.docm]

**Supplementary materials**

**Relationship Between Internet Use Purposes and Depressive Symptoms Among Chinese Older Adults: A Cross-Lagged Panel Network Analysis**

Table S1. Missing frequency and Percentage for a dataset of 9290 participants.

Table S2. Correlation matrix of missing data patterns.

Table S3. Adjacency matrix of the temporal network (including autoregressive edges).

Table S4. Adjacency matrix of the temporal network.

Table S5. Multicollinearity diagnostics of variables.

Figure S1. Bootstrapped 95% confidence intervals around temporal network edges.

Figure S2. Centrality of the temporal network.

Figure S3. Centrality of difference tests for the temporal network.

Figure S4. Edge weight difference tests for the temporal network.

Figure S5. The cross-lagged panel network analysis of internet use purpose and depressive symptoms (including autoregressive edges).

Table S1. Missing frequency and Percentage for a dataset of 9290 participants.

| Variable | N (%) (T1) | N (%) (T2) |
| --- | --- | --- |
| Depressive symptoms |  | |
| Unusually bothered | 1054(11.35) | 1115(12.00) |
| Mind adrift | 1293(13.92) | 1258(13.54) |
| Felt depressed | 1055(11.36) | 1233(13.27) |
| Everything was an effort | 2511(27.03) | 1145(12.33) |
| Felt hopeful about the future | 1422(15.31) | 1435(15.45) |
| Felt fearful | 901(9.70) | 986(10.61) |
| Restless sleep | 880(9.47) | 972(10.46) |
| Lack of happiness | 948(10.20) | 1064(11.45) |
| Loneliness | 979(10.54) | 1100(11.84) |
| Inability get going | 1090(11.73) | 1154(12.42) |
| Internet use purposes |  | |
| Chatting | 0(0) | 0(0) |
| Watching news | 0(0) | 0(0) |
| Watching videos | 0(0) | 0(0) |
| Playing games | 0(0) | 0(0) |
| Financial management | 0(0) | 0(0) |
| Gender | 0(0) | - |
| Age | 0(0) | - |
| Education level | 1(0.01) | - |
| Chronic diseases history | 0(0) | - |
| Types of devices for accessing the Internet | 0(0) | - |
| The frequency of Internet use | 0(0) | - |

Note: - represents Unapplicable, N represents frequency, % represents proportion, T1 represents the data of 2018, T2 represents the data of 2020.

Table S2. Correlation matrix of missing data patterns.

| Variable | De1T1 | De2T1 | De3T1 | De4T1 | De5T1 | De6T1 | De7T1 | De8T1 | De9T1 | De10T1 | De1T2 | De2T2 | De3T2 | De4T2 | De5T2 | De6T2 | De7T2 | De8T2 | De9T2 | De10T2 | Educ |
| --- | --- | --- | --- | --- | --- | --- | --- | --- | --- | --- | --- | --- | --- | --- | --- | --- | --- | --- | --- | --- | --- |
| De1T1 | 1.0000 | 0.7769 | 0.8165 | 0.4938 | 0.7123 | 0.8266 | 0.8277 | 0.8145 | 0.8102 | 0.7829 | 0.4235 | 0.4020 | 0.3953 | 0.4121 | 0.3749 | 0.4321 | 0.4333 | 0.4288 | 0.4215 | 0.4076 | -0.0037 |
| De2T1 | 0.7769 | 1.0000 | 0.7882 | 0.5122 | 0.7161 | 0.7635 | 0.7577 | 0.7552 | 0.7583 | 0.7328 | 0.4094 | 0.3989 | 0.3936 | 0.4008 | 0.3832 | 0.4208 | 0.4193 | 0.4218 | 0.4099 | 0.4058 | -0.0042 |
| De3T1 | 0.8165 | 0.7882 | 1.0000 | 0.5239 | 0.7468 | 0.8434 | 0.8435 | 0.8331 | 0.8462 | 0.8025 | 0.4159 | 0.4007 | 0.4030 | 0.4097 | 0.3840 | 0.4307 | 0.4263 | 0.4253 | 0.4128 | 0.4052 | -0.0037 |
| De4T1 | 0.4938 | 0.5122 | 0.5239 | 1.0000 | 0.5221 | 0.5123 | 0.5025 | 0.4914 | 0.5087 | 0.5057 | 0.2876 | 0.2791 | 0.2827 | 0.2865 | 0.2777 | 0.2868 | 0.2869 | 0.2880 | 0.2826 | 0.2903 | -0.0063 |
| De5T1 | 0.7123 | 0.7161 | 0.7468 | 0.5221 | 1.0000 | 0.7406 | 0.7303 | 0.7288 | 0.7372 | 0.7266 | 0.3821 | 0.3770 | 0.3756 | 0.3863 | 0.3767 | 0.3903 | 0.3909 | 0.3878 | 0.3892 | 0.3874 | -0.0044 |
| De6T1 | 0.8266 | 0.7635 | 0.8434 | 0.5123 | 0.7406 | 1.0000 | 0.9225 | 0.8941 | 0.8909 | 0.8424 | 0.4274 | 0.4146 | 0.4089 | 0.4259 | 0.3903 | 0.4515 | 0.4501 | 0.4418 | 0.4327 | 0.4202 | -0.0034 |
| De7T1 | 0.8277 | 0.7577 | 0.8435 | 0.5025 | 0.7303 | 0.9225 | 1.0000 | 0.9049 | 0.8862 | 0.8415 | 0.4257 | 0.4113 | 0.4076 | 0.4211 | 0.3896 | 0.4506 | 0.4479 | 0.4435 | 0.4298 | 0.4187 | -0.0034 |
| De8T1 | 0.8145 | 0.7552 | 0.8331 | 0.4914 | 0.7288 | 0.8941 | 0.9049 | 1.0000 | 0.8791 | 0.8384 | 0.4259 | 0.4122 | 0.4037 | 0.4199 | 0.3873 | 0.4472 | 0.4459 | 0.4438 | 0.4279 | 0.4196 | -0.0035 |
| De9T1 | 0.8102 | 0.7583 | 0.8462 | 0.5087 | 0.7372 | 0.8909 | 0.8862 | 0.8791 | 1.0000 | 0.8531 | 0.4331 | 0.4205 | 0.4144 | 0.4291 | 0.3965 | 0.4474 | 0.4485 | 0.4479 | 0.4417 | 0.4256 | -0.0036 |
| De10T1 | 0.7829 | 0.7328 | 0.8025 | 0.5057 | 0.7266 | 0.8424 | 0.8415 | 0.8384 | 0.8531 | 1.0000 | 0.4160 | 0.4071 | 0.3976 | 0.4107 | 0.3875 | 0.4336 | 0.4327 | 0.4340 | 0.4223 | 0.4134 | -0.0038 |
| De1T2 | 0.4235 | 0.4094 | 0.4159 | 0.2876 | 0.3821 | 0.4274 | 0.4257 | 0.4259 | 0.4331 | 0.4160 | 1.0000 | 0.8596 | 0.8503 | 0.8641 | 0.7788 | 0.8900 | 0.8931 | 0.8677 | 0.8601 | 0.8390 | -0.0038 |
| De2T2 | 0.4020 | 0.3989 | 0.4007 | 0.2791 | 0.3770 | 0.4146 | 0.4113 | 0.4122 | 0.4205 | 0.4071 | 0.8596 | 1.0000 | 0.8494 | 0.8565 | 0.7823 | 0.8390 | 0.8401 | 0.8287 | 0.8267 | 0.8229 | -0.0041 |
| De3T2 | 0.3953 | 0.3936 | 0.4030 | 0.2827 | 0.3756 | 0.4089 | 0.4076 | 0.4037 | 0.4144 | 0.3976 | 0.8503 | 0.8494 | 1.0000 | 0.8764 | 0.8099 | 0.8520 | 0.8510 | 0.8476 | 0.8592 | 0.8386 | -0.0041 |
| De4T2 | 0.4121 | 0.4008 | 0.4097 | 0.2865 | 0.3863 | 0.4259 | 0.4211 | 0.4199 | 0.4291 | 0.4107 | 0.8641 | 0.8565 | 0.8764 | 1.0000 | 0.8102 | 0.8957 | 0.8925 | 0.8749 | 0.8721 | 0.8685 | -0.0039 |
| De5T2 | 0.3749 | 0.3832 | 0.3840 | 0.2777 | 0.3767 | 0.3903 | 0.3896 | 0.3873 | 0.3965 | 0.3875 | 0.7788 | 0.7823 | 0.8099 | 0.8102 | 1.0000 | 0.7898 | 0.7871 | 0.7947 | 0.7920 | 0.7899 | -0.0044 |
| De6T2 | 0.4321 | 0.4208 | 0.4307 | 0.2868 | 0.3903 | 0.4515 | 0.4506 | 0.4472 | 0.4474 | 0.4336 | 0.8900 | 0.8390 | 0.8520 | 0.8957 | 0.7898 | 1.0000 | 0.9544 | 0.9164 | 0.9002 | 0.8736 | -0.0036 |
| De7T2 | 0.4333 | 0.4193 | 0.4263 | 0.2869 | 0.3909 | 0.4501 | 0.4479 | 0.4459 | 0.4485 | 0.4327 | 0.8931 | 0.8401 | 0.8510 | 0.8925 | 0.7871 | 0.9544 | 1.0000 | 0.9229 | 0.9034 | 0.8821 | -0.0035 |
| De8T2 | 0.4288 | 0.4218 | 0.4253 | 0.2880 | 0.3878 | 0.4418 | 0.4435 | 0.4438 | 0.4479 | 0.4340 | 0.8677 | 0.8287 | 0.8476 | 0.8749 | 0.7947 | 0.9164 | 0.9229 | 1.0000 | 0.8977 | 0.8730 | -0.0037 |
| De9T2 | 0.4215 | 0.4099 | 0.4128 | 0.2826 | 0.3892 | 0.4327 | 0.4298 | 0.4279 | 0.4417 | 0.4223 | 0.8601 | 0.8267 | 0.8592 | 0.8721 | 0.7920 | 0.9002 | 0.9034 | 0.8977 | 1.0000 | 0.8832 | -0.0038 |
| De10T2 | 0.4076 | 0.4058 | 0.4052 | 0.2903 | 0.3874 | 0.4202 | 0.4187 | 0.4196 | 0.4256 | 0.4134 | 0.8390 | 0.8229 | 0.8386 | 0.8685 | 0.7899 | 0.8736 | 0.8821 | 0.8730 | 0.8832 | 1.0000 | -0.0039 |
| Educ | -0.0037 | -0.0042 | -0.0037 | -0.0063 | -0.0044 | -0.0034 | -0.0034 | -0.0035 | -0.0036 | -0.0038 | -0.0038 | -0.0041 | -0.0041 | -0.0039 | -0.0044 | -0.0036 | -0.0035 | -0.0037 | -0.0038 | -0.0039 | 1.0000 |

Note: De represents depressive symptoms, Educ represents education level, T1 represents the data of 2018, T2 represents the data of 2020.

The correlation matrix shows relationships between missing patterns of variables. Missing values are encoded as 1, non-missing as 0, and pairwise correlations are calculated using the pairwise.complete.obs method. High values indicate strong dependencies in missingness.

**Table S3.** Adjacency matrix of the temporal network (including autoregressive edges).

|  | De1T2 | De2T2 | De3T2 | De4T2 | De5T2 | De6T2 | De7T2 | De8T2 | De9T2 | De10T2 | In1T2 | In2T2 | In3T2 | In4T2 | In5T2 |
| --- | --- | --- | --- | --- | --- | --- | --- | --- | --- | --- | --- | --- | --- | --- | --- |
| De1T1 | 0.13 | 0.04 | 0.07 | 0.03 | 0.00 | 0.03 | 0.03 | 0.04 | 0.05 | 0.05 | 0.00 | 0.00 | 0.00 | 0.00 | 0.00 |
| De2T1 | 0.01 | 0.09 | 0.03 | 0.07 | 0.00 | 0.00 | 0.00 | 0.00 | -0.01 | -0.03 | 0.00 | 0.00 | 0.00 | 0.00 | 0.00 |
| De3T1 | 0.09 | 0.06 | 0.13 | 0.07 | -0.01 | 0.03 | 0.02 | 0.03 | 0.03 | 0.06 | 0.00 | 0.00 | 0.00 | 0.00 | 0.00 |
| De4T1 | 0.04 | 0.06 | 0.08 | 0.17 | 0.03 | 0.05 | 0.03 | 0.05 | 0.05 | 0.08 | -0.01 | -0.01 | 0.00 | 0.00 | 0.00 |
| De5T1 | 0.01 | 0.02 | 0.02 | 0.06 | 0.20 | 0.01 | 0.01 | 0.08 | 0.03 | 0.03 | -0.01 | -0.01 | -0.01 | 0.00 | 0.00 |
| De6T1 | 0.05 | 0.07 | 0.04 | 0.02 | -0.04 | 0.19 | 0.01 | 0.00 | 0.05 | 0.07 | 0.00 | 0.00 | -0.01 | 0.00 | 0.00 |
| De7T1 | 0.06 | 0.02 | 0.03 | 0.05 | 0.00 | 0.02 | 0.34 | 0.01 | 0.02 | 0.02 | 0.00 | 0.00 | 0.01 | 0.00 | 0.00 |
| De8T1 | 0.07 | 0.04 | 0.06 | 0.04 | 0.04 | 0.02 | 0.05 | 0.19 | 0.05 | 0.04 | 0.00 | 0.00 | 0.00 | 0.00 | 0.00 |
| De9T1 | 0.04 | 0.06 | 0.05 | 0.05 | 0.01 | 0.02 | 0.02 | 0.06 | 0.21 | 0.05 | 0.00 | 0.00 | 0.00 | 0.00 | 0.00 |
| De10T1 | 0.04 | 0.00 | 0.04 | 0.04 | 0.05 | 0.04 | 0.00 | 0.05 | 0.07 | 0.13 | -0.01 | -0.01 | -0.02 | 0.00 | 0.00 |
| In1T1 | 0.00 | 0.00 | 0.00 | 0.00 | -0.05 | 0.00 | 0.06 | -0.09 | 0.00 | -0.02 | 0.29 | 0.02 | 0.09 | 0.00 | 0.00 |
| In2T1 | -0.05 | 0.00 | -0.09 | -0.19 | -0.16 | -0.02 | 0.00 | -0.02 | -0.09 | -0.06 | 0.11 | 0.38 | 0.13 | 0.00 | 0.00 |
| In3T1 | 0.00 | 0.00 | 0.00 | 0.09 | -0.03 | 0.00 | 0.00 | -0.01 | 0.02 | 0.00 | 0.00 | 0.01 | 0.10 | -0.01 | -0.03 |
| In4T1 | 0.00 | 0.00 | -0.03 | -0.12 | 0.06 | 0.00 | 0.00 | 0.00 | 0.00 | -0.05 | -0.01 | 0.00 | 0.06 | 0.44 | 0.00 |
| In5T1 | 0.09 | -0.02 | -0.06 | 0.00 | -0.04 | 0.00 | 0.00 | 0.14 | -0.01 | -0.02 | 0.00 | 0.00 | 0.00 | -0.03 | 0.45 |

Note. De represents depressive symptoms, In represents Internet use purposes, T1 represents the data of 2018, T2 represents the data of 2020. The rows present independent variables, and The columns present dependent variables. Autoregressive edges are presented along the diagonal. The results adjusted the covariates, including age, gender, education level, chronic diseases history, types of devices for accessing the Internet, and the frequency of Internet use.

**Table S4.** Adjacency matrix of the temporal network.

|  | De1T2 | De2T2 | De3T2 | De4T2 | De5T2 | De6T2 | De7T2 | De8T2 | De9T2 | De10T2 | In1T2 | In2T2 | In3T2 | In4T2 | In5T2 |
| --- | --- | --- | --- | --- | --- | --- | --- | --- | --- | --- | --- | --- | --- | --- | --- |
| De1T1 | 0.00 | 0.04 | 0.07 | 0.03 | 0.00 | 0.03 | 0.03 | 0.04 | 0.05 | 0.05 | 0.00 | 0.00 | 0.00 | 0.00 | 0.00 |
| De2T1 | 0.01 | 0.00 | 0.03 | 0.07 | 0.00 | 0.00 | 0.00 | 0.00 | -0.01 | -0.03 | 0.00 | 0.00 | 0.00 | 0.00 | 0.00 |
| De3T1 | 0.09 | 0.06 | 0.00 | 0.07 | -0.01 | 0.03 | 0.02 | 0.03 | 0.03 | 0.06 | 0.00 | 0.00 | 0.00 | 0.00 | 0.00 |
| De4T1 | 0.04 | 0.06 | 0.08 | 0.00 | 0.03 | 0.05 | 0.03 | 0.05 | 0.05 | 0.08 | -0.01 | -0.01 | 0.00 | 0.00 | 0.00 |
| De5T1 | 0.01 | 0.02 | 0.02 | 0.06 | 0.00 | 0.01 | 0.01 | 0.08 | 0.03 | 0.03 | -0.01 | -0.01 | -0.01 | 0.00 | 0.00 |
| De6T1 | 0.05 | 0.07 | 0.04 | 0.02 | -0.04 | 0.00 | 0.01 | 0.00 | 0.05 | 0.07 | 0.00 | 0.00 | -0.01 | 0.00 | 0.00 |
| De7T1 | 0.06 | 0.02 | 0.03 | 0.05 | 0.00 | 0.02 | 0.00 | 0.01 | 0.02 | 0.02 | 0.00 | 0.00 | 0.01 | 0.00 | 0.00 |
| De8T1 | 0.07 | 0.04 | 0.06 | 0.04 | 0.04 | 0.02 | 0.05 | 0.00 | 0.05 | 0.04 | 0.00 | 0.00 | 0.00 | 0.00 | 0.00 |
| De9T1 | 0.04 | 0.06 | 0.05 | 0.05 | 0.01 | 0.02 | 0.02 | 0.06 | 0.00 | 0.05 | 0.00 | 0.00 | 0.00 | 0.00 | 0.00 |
| De10T1 | 0.04 | 0.00 | 0.04 | 0.04 | 0.05 | 0.04 | 0.00 | 0.05 | 0.07 | 0.00 | -0.01 | -0.01 | -0.02 | 0.00 | 0.00 |
| In1T1 | 0.00 | 0.00 | 0.00 | 0.00 | -0.05 | 0.00 | 0.06 | -0.09 | 0.00 | -0.02 | 0.00 | 0.02 | 0.09 | 0.00 | 0.00 |
| In2T1 | -0.05 | 0.00 | -0.09 | -0.19 | -0.16 | -0.02 | 0.00 | -0.02 | -0.09 | -0.06 | 0.11 | 0.00 | 0.13 | 0.00 | 0.00 |
| In3T1 | 0.00 | 0.00 | 0.00 | 0.09 | -0.03 | 0.00 | 0.00 | -0.01 | 0.02 | 0.00 | 0.00 | 0.01 | 0.00 | -0.01 | -0.03 |
| In4T1 | 0.00 | 0.00 | -0.03 | -0.12 | 0.06 | 0.00 | 0.00 | 0.00 | 0.00 | -0.05 | -0.01 | 0.00 | 0.06 | 0.00 | 0.00 |
| In5T1 | 0.09 | -0.02 | -0.06 | 0.00 | -0.04 | 0.00 | 0.00 | 0.14 | -0.01 | -0.02 | 0.00 | 0.00 | 0.00 | -0.03 | 0.00 |

Note. De represents depressive symptoms, In represents Internet use purposes, T1 represents the data of 2018, T2 represents the data of 2020. The rows present independent variables, and The columns present dependent variables. Autoregressive edges are excluded. The results adjusted the covariates, including age, gender, education level, chronic diseases history, types of devices for accessing the Internet, and the frequency of Internet use.

**Table S5.** Multicollinearity diagnostics of variables

| Variables | Tolerance | VIF |
| --- | --- | --- |
| De1T1 | 0.588 | 1.700 |
| De2T1 | 0.635 | 1.576 |
| De3T1 | 0.507 | 1.972 |
| De4T1 | 0.617 | 1.620 |
| De5T1 | 0.772 | 1.295 |
| De6T1 | 0.729 | 1.373 |
| De7T1 | 0.797 | 1.255 |
| De8T1 | 0.750 | 1.334 |
| De9T1 | 0.652 | 1.535 |
| De10T1 | 0.637 | 1.571 |
| In1T1 | 0.435 | 2.297 |
| In2T1 | 0.209 | 4.788 |
| In3T1 | 0.343 | 2.913 |
| In4T1 | 0.726 | 1.377 |
| In5T1 | 0.901 | 1.110 |
| Gender | 0.851 | 1.175 |
| Age | 0.960 | 1.041 |
| Education level | 0.922 | 1.085 |
| Chronic diseases history | 0.206 | 4.850 |
| Types of devices for accessing the Internet | 0.277 | 3.609 |
| The frequency of Internet use | 0.823 | 1.215 |

Note. De represents depressive symptoms, In represents Internet use purposes, T1 represents the data of 2018, T2 represents the data of 2020.


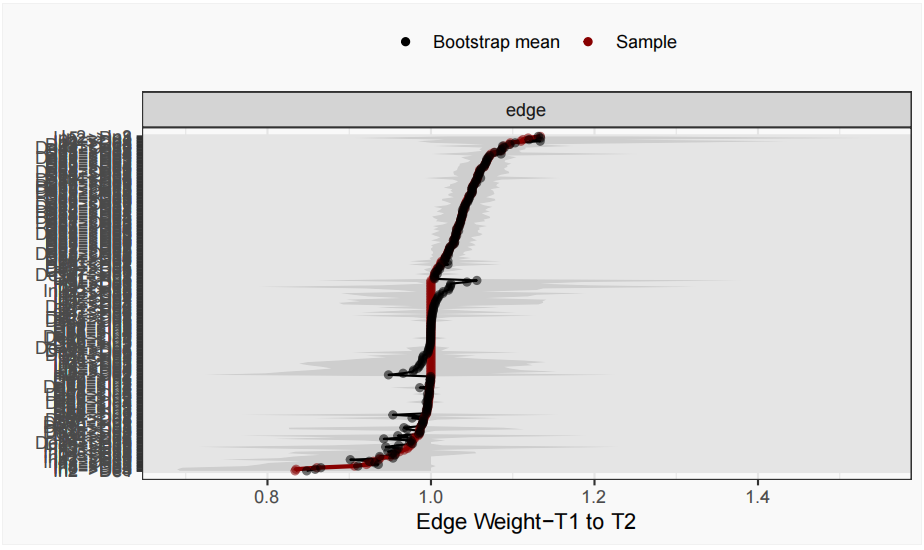


**Figure S1.** Bootstrapped 95% confidence intervals around temporal network edges.

Note. Red lines indicate the edge weight in the estimated sample network.


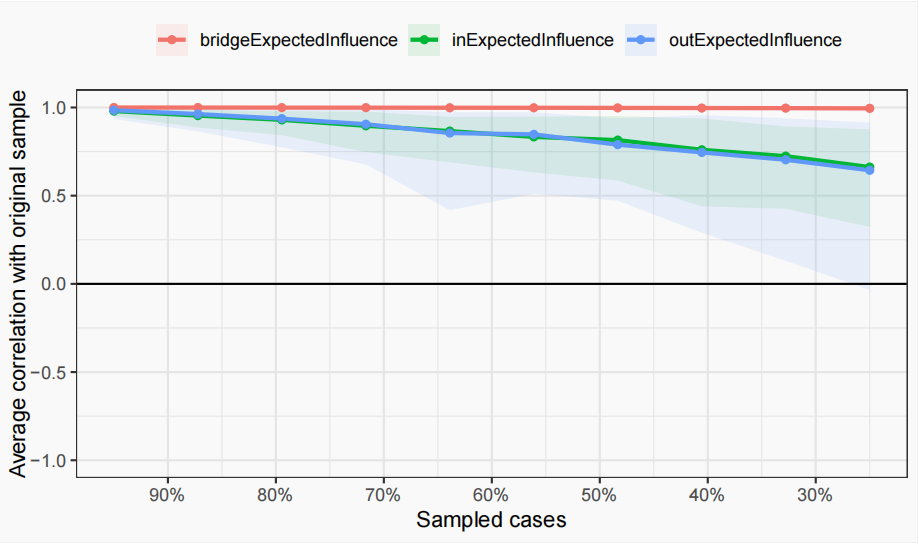


**Figure S2.** Centrality of the temporal network.


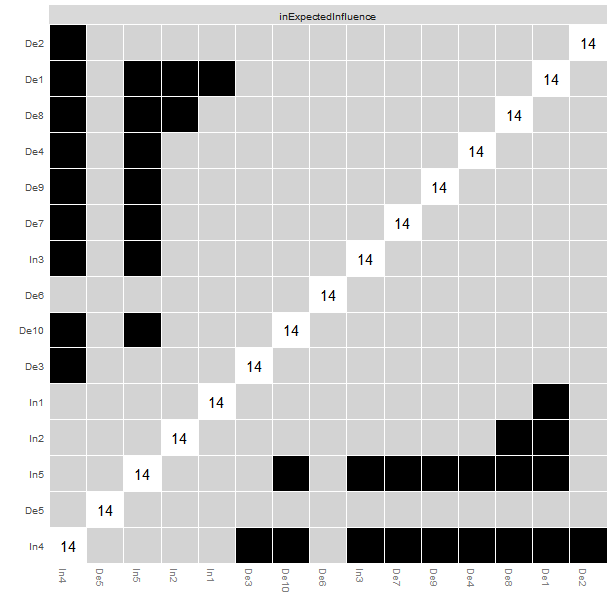

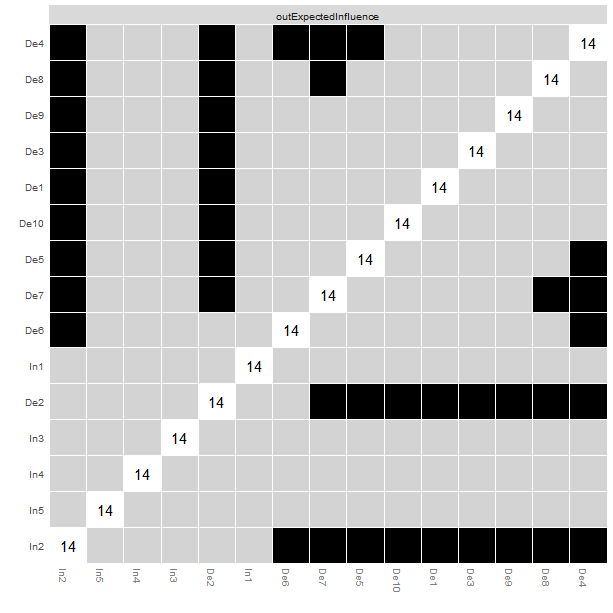

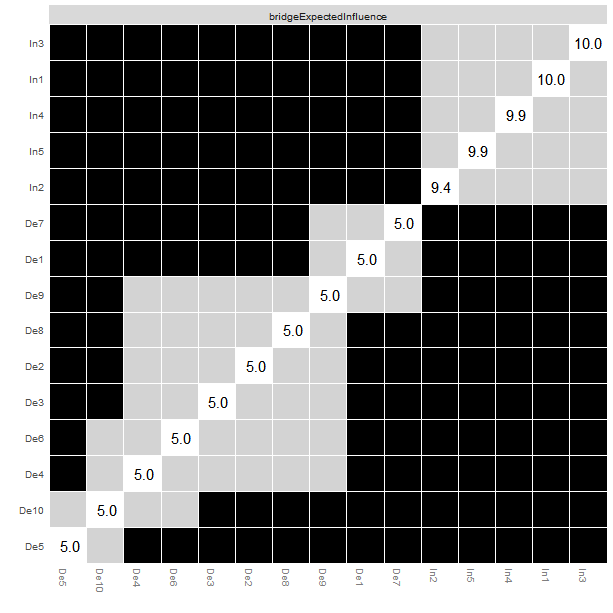


**Figure S3.** Centrality of difference tests for the temporal network.

Note. Grey boxes represent non-significant differences between node pairs, and black boxes represent significant differences between node pairs (p < 0.05). The white boxes in the centrality plot show the Out-Expected Influence, In-Expected Influence, and Bridge-Expected Influence values. de, depressive symptoms; in, internet use purposes.


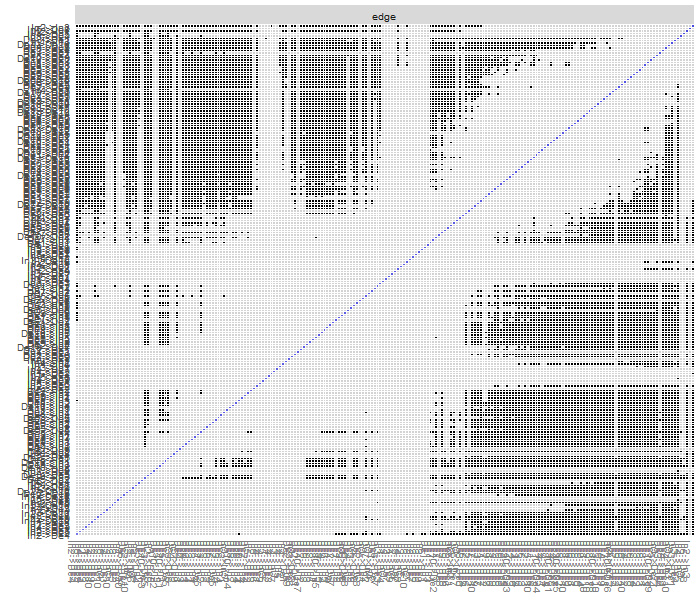


**Figure S4**. Edge weight difference tests for the temporal network.

Note. Grey boxes represent non-significant differences between node pairs, and black boxes represent significant differences between node pairs (p < 0.05).


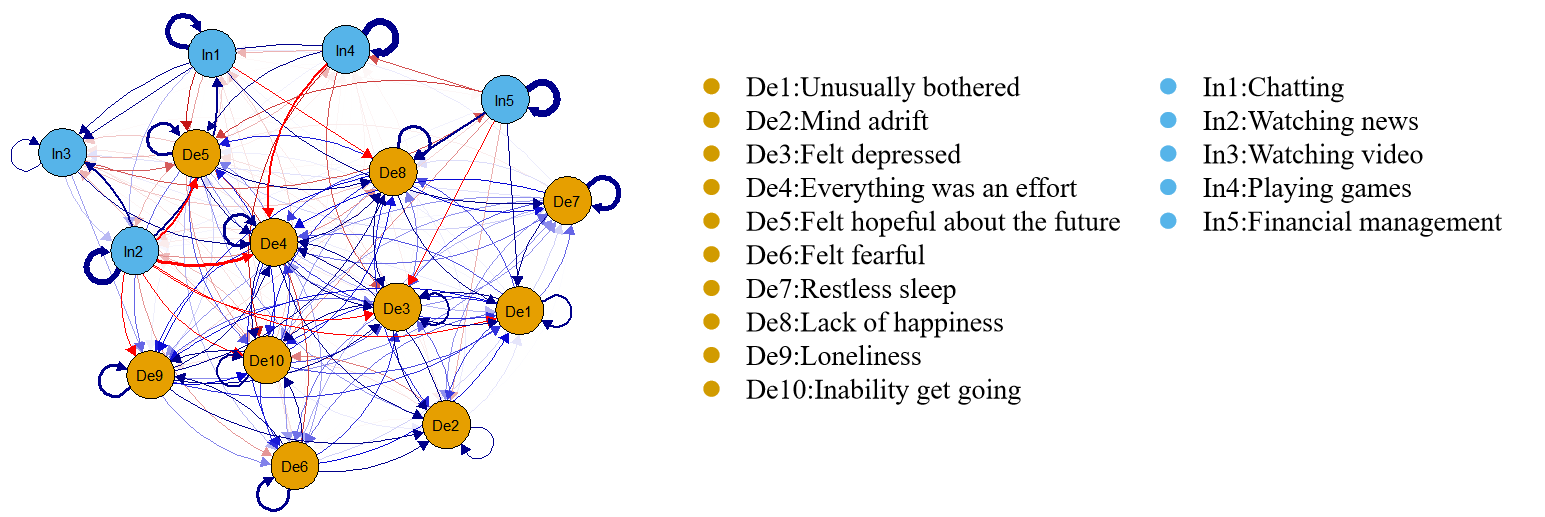


**Figure S5.** The cross-lagged panel network analysis of internet use purpose and depressive symptoms (including autoregressive edges).

Note. |β| < 0.05 were excluded in order to visual demonstration of the temporal relationships.
